# Clinker: visualizing fusion genes detected in RNA-seq data

**DOI:** 10.1093/gigascience/giy079

**Published:** 2018-07-04

**Authors:** Breon M Schmidt, Nadia M Davidson, Anthony D K Hawkins, Ray Bartolo, Ian J Majewski, Paul G Ekert, Alicia Oshlack

**Affiliations:** 1 Murdoch Children's Research Institute, The Royal Children's Hospital, Flemington, Road, Parkville Vic 3052 Australia; 2 School of Biosciences, University of Melbourne, Parkivlle Vic 3010, Australia; 3 Division of Cancer and Haematology, The Walter and Eliza Hall Institute of Medical Research, Parkville Vic 3052, Australia; 4 Faculty of Medicine, Dentistry and Health Sciences, University of Melbourne, Parkville Vic 3010, Australia

**Keywords:** cancer, RNA-seq, fusion genes, visualization

## Abstract

**Background:**

Genomic profiling efforts have revealed a rich diversity of oncogenic fusion genes. While there are many methods for identifying fusion genes from RNA-sequencing (RNA-seq) data, visualizing these transcripts and their supporting reads remains challenging.

**Findings:**

Clinker is a bioinformatics tool written in Python, R, and Bpipe that leverages the superTranscript method to visualize fusion genes. We demonstrate the use of Clinker to obtain interpretable visualizations of the RNA-seq data that lead to fusion calls. In addition, we use Clinker to explore multiple fusion transcripts with novel breakpoints within the *P2RY8-CRLF2* fusion gene in B-cell acute lymphoblastic leukemia.

**Conclusions:**

Clinker is freely available software that allows visualization of fusion genes and the RNA-seq data used in their discovery.

## Introduction

Genomic structural abnormalities, such as translocations between and within chromosomes, are common in cancer and can result in the fusion of two genes that then function as an oncogenic driver. The first example of this was the recurrent t(9;22) fusion in chronic myeloid leukemia, creating the *BCR-ABL1* oncogene [1]. This fusion gene results in a constitutively activated tyrosine kinase protein that can be effectively treated with small molecule inhibitors of ABL1, such as imatinib and dasatinib [2]. The application of next-generation sequencing in cancer, primarily transcriptome sequencing (RNA-sequencing [RNA-seq]), has subsequently identified thousands of different fusion genes in many cancer types [3].

While there are many methods available for identifying fusion genes from RNA-seq data, there are few ways to visualize the fusion transcripts and the sequencing reads that support them. Simply aligning RNA-seq data to a reference genome or transcriptome does not allow clear visualization of the translocation or an appreciation of additional features such as splice variants. One approach for visualizing the translocation is to use the split screen view within IGV. However, because RNA-seq read coverage is sparse in the genome, visualization is hampered by the presence of introns. Other strategies that address this problem involve using predicted breakpoints to create the fusion transcript sequence, which can be used as a reference for read alignment [4, 5]. This approach demonstrates coverage across the fusion breakpoints. However, other information about the structure and expression of the fusion transcripts, such as its expression relative to nonfused transcripts, can be lost. In addition, intronic sequence can be “shrunk” to give a more informative view of coverage [6].

Here, we provide an alternative tool, Clinker, for visualizing RNA-seq data of fusion genes that enables a greater understanding of transcript coverage and splicing isoforms. Clinker, utilizes superTranscripts, a new type of transcriptome reference we previously developed that contains only the transcribed sequence of a gene, without introns, providing a highly compact reference for analysis and visualization of RNA-seq [7]. Clinker uses the human superTranscript references and creates fusion-superTranscripts by combining the two genes involved in a fusion event.

We applied Clinker to a set of six B-cell acute lymphoblastic leukemias (ALLs) that all report the *P2RY8-CRLF2* fusion to demonstrate several fusion isoforms. Clinker is a tool that provides direct visualization of fusion genes and allows further appreciation of their complexity, such as alternative fusion isoforms.

## Materials and Methods

### Reference and annotation generation

The Clinker pipeline takes output from any fusion calling software, providing that it includes the hg19 or hg38 genomic coordinates of fusion gene breakpoints. These breakpoints are used to identify the two genes involved in the fusion and assigns them consistent gene symbols. This method is preferred even when gene symbols are provided by the fusion caller; this is due to the large variability in gene naming conventions. Once the two genes are identified, their sequences are retrieved from Clinker's human superTranscript reference and concatenated to form a single fusion-superTranscript. An important feature of the fusion-superTranscript reference is that it includes the full sequence of both genes orientated in transcriptional direction. Thus, reads aligned to regions of the genes not involved in the fusion are also visualized, providing additional information about expression of these regions and the domains they encode. This is repeated for all fusion genes that have been identified in the sample. This results in a sample-specific Clinker reference containing the fusion-superTranscripts as well as the superTranscripts from all normal genes. We found it was important to map competitively to the nonfused genes in the reference to avoid spurious read alignments. Transcript, protein domain, and gene boundary annotation files are also created using the Gencode24 hg38 reference [8] and the Pfam protein database [9] to provide additional information for the visualization.

### Alignment to the new reference

Clinker maps the sequencing reads to the newly generated reference using the STAR aligner (STAR, RRID:SCR_015899) [10]. The aligner must be splice aware as reads spanning the fusion breakpoint are identified as splice sites. The alignment stage of Clinker often yields greater read support for fusion genes than fusion callers. For example, in one sample, JAFFA detected the *P2RY8-CRLF2* fusion with support from 53 spanning reads, whereas Clinker, through STAR, reported 290 spanning reads (Supplementary Table S1). As Clinker is given prior knowledge that a fusion exists between two genes, the fusion-superTranscript can be mapped to with less stringency, leading to the increase in successfully mapped reads across the breakpoint. After the alignment step, the mapped reads and fusion genes can be viewed with a genome viewer, such as IGV, by loading the Clinker reference FASTA, mapped reads, and the customized transcript, protein domain, and gene boundary annotation tracks. IGV natively displays the fusion breakpoints and splice junctions through the splice junction track or sashimi plot (Fig.2B).

### Filtering, normalization, and figure creation

Once reads are aligned, filtering and normalization steps are undertaken. Split reads with a small number of flanking bases on one side can be produced by incorrect split-read alignment. To account for this, split reads with fewer than 5 base pairs of flanking sequence are immediately filtered out using both Samtools (SAMTOOLS, RRID:SCR_002105) [11] and custom AWK scripting (see Supplementary Fig. S2 for an example). Coverage is normalized to reads per million using STAR's inbuilt normalization function to allow comparison between samples.

A series of figures, one for each of the identified fusion genes, is then created using the R package, GViz [12]. These figures contain multiple tracks including coverage, gene boundaries, protein domains, and the transcripts/exons involved in the fusion gene. A sashimi plot is also included in the figure to indicate the number of split reads that support the fusion, with three reads being set as a minimum threshold to further filter out spurious splicing events. The order, color, or presence of the tracks can be customized via the command line parameters of Clinker.

### Software requirements

Clinker can be run both manually and through Bpipe (Bpipe, RRID:SCR_003471) [13], a tool for running bioinformatics pipelines. The core dependencies for Clinker are STAR [10], Samtools [11], and Gviz [12]. Runtime was approximately 1 hour with eight processors and 40 GB of memory allocated for a single publication-quality figure and an additional 1 minute for each additional figure. This test was conducted on a sample with approximately 130 million reads and 2,007 fusion genes reported by JAFFA.

### *P2RY8-CRLF2* cloning and expression

We applied Clinker to a set of six B-cell ALL patient samples for which the *P2RY8-CRLF2* fusion gene was detected (Supplementary Fig. S1). We also found that when using the JAFFA fusion caller, several noncanonical fusion isoforms were reported. RNA-seq data for these sample can be found at [14].

Fusion detection by RNA-seq was confirmed by polymerase chain reaction (PCR) using gene-specific primers for *P2YR8* (5′-CAAGGTTGCTGGACAGATGGAA-3′) and *CRLF2* (5′-AATAGAGAATGTCGTCTCGCTGC-3′). Primers were designed to amplify products spanning the exons at the breakpoints of *P2RY8* and *CRLF2* in the mRNA transcripts detected by JAFFA (Supplementary Fig. S3). The alternate and frameshift fusions were cloned using primers to target the start of P2RY8 (5- CCCTGCACATGAGTGTTCAGAC-3′) and the end of *CRLF2* (5′-TCACAACGCCACGTAGGAG-3′), while the canonical fusion was amplified using a different *P2RY8* forward primer (5′-GCGGCCGCCTTTGCAAGGTTGC-3′) (Supplementary Fig. S4). PCR products were cloned into P-GEM-T easy vector (Promega), Sanger sequenced, and then subcloned into a retroviral pMSCV-GFP retroviral expression vector. Retrovirus was produced as previously described [15] and transduced into IL3-dependant BaF3 cells. *CRLF2* was detected in the BaF3 cells using the Anti-Human TSLP receptor antibody (eBioscience) and the BD Cytofix/Cytoperm (BD Biosciences), according the manufacturer's instructions (Supplementary Fig. S5). FACs analysis was performed on an LSRII flow cytometer (BD Biosciences).

## Results

### The Clinker pipeline

**Figure 1.**
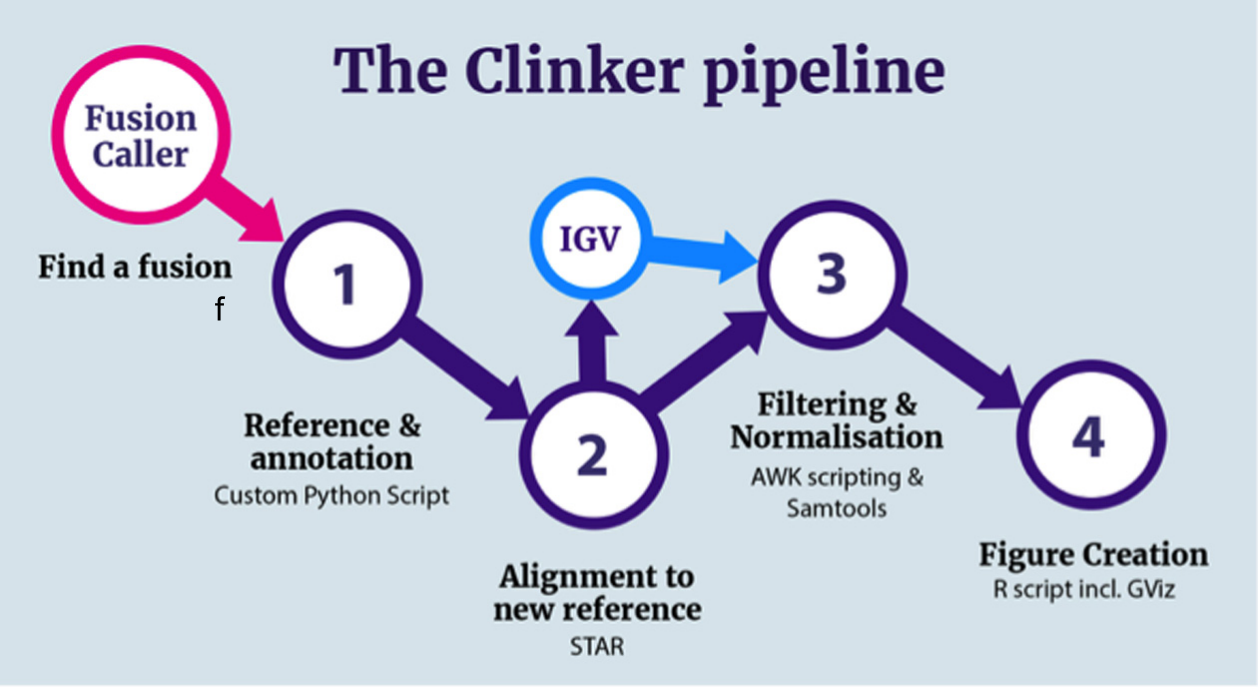
: A visual representation of the Clinker pipeline. Users can choose to stop at step 2, inspect fusion genes of interest in IGV, and then commence figure production for a refined list of fusion genes. The Fusion Caller (pink) and IGV (light blue) steps are external to Clinker.

Clinker is an analysis pipeline that takes in fusion calls and raw RNA-seq reads and outputs a custom reference, mapped read data, and image files to visualize and assess fusion transcripts. The steps in this pipeline are outlined in Fig.1 and described in detail in the Materials and Methods section.

Briefly, before running Clinker, fusions are detected using one of the many specialized fusion gene callers, such as JAFFA [16], STAR-fusion [17], or Pizzly [18]. Clinker proceeds by first concatenating the full-length superTranscripts of the two genes involved in the fusion for each event called in the sample. These fused superTranscripts are then added to a custom, sample-specific superTranscriptome reference. Next, the reads are mapped back to the new reference using the STAR splice aware aligner [10]. A fusion can then be observed as splicing between the two concatenated genes. Finally, figures are generated that present the resulting splice junctions, coverage, protein domains, and transcript annotation for both the fusion and nonfused superTranscripts. Clinker outputs file formats that are compatible with IGV [19], as well as publication-quality images created with Gviz [12].

### Clinker visualizations of reads, transcripts, and protein domains

**Figure 2.**
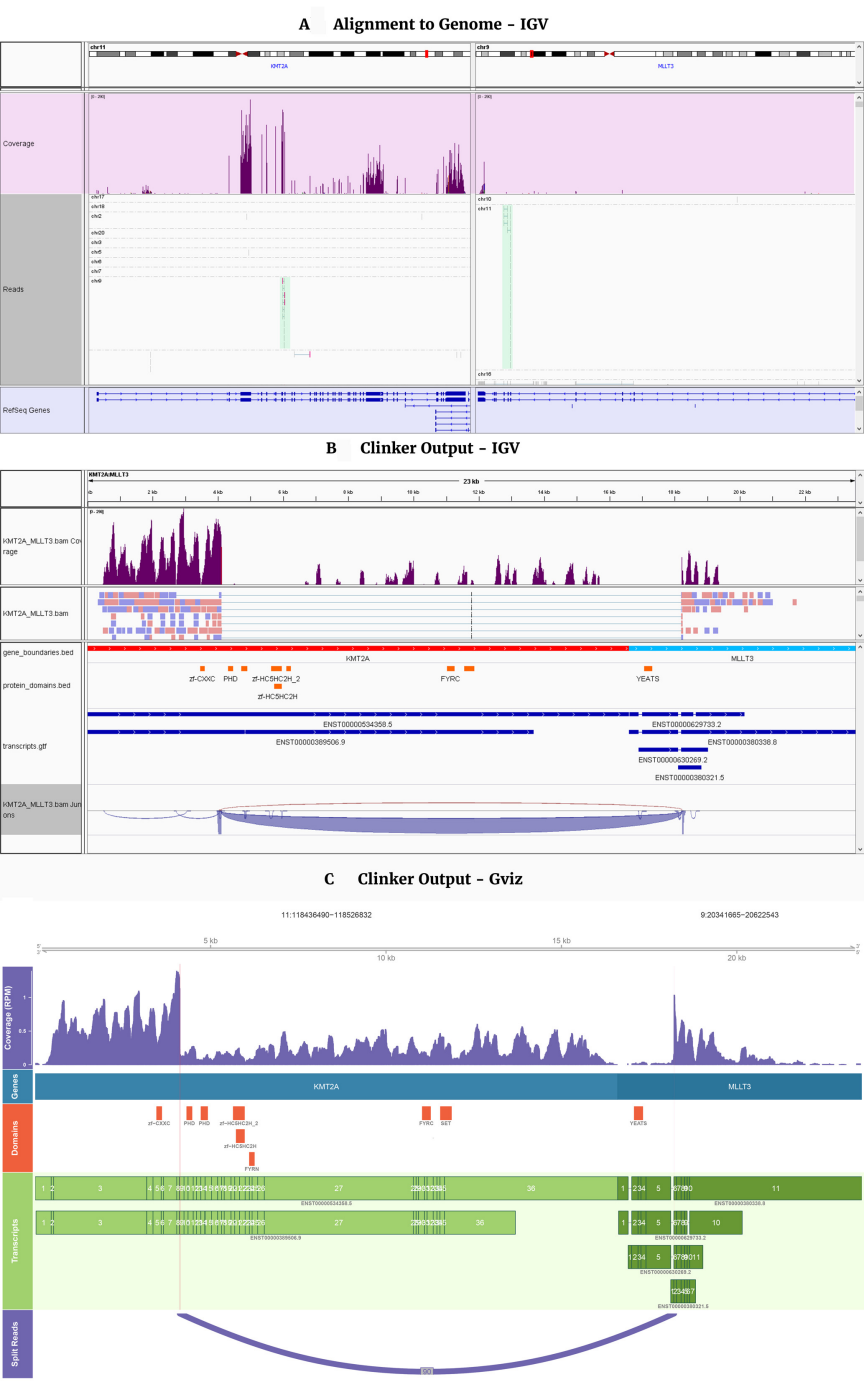
: **(A)***KMT2A-MLLT3* fusion gene visualized in IGV after alignment to the human genome. The backgrounds of the IGV tracks are colored to distinguish between the coverage (purple), aligned reads (white), and annotation (blue), with green reads indicating that its partner is on a different chromosome. Such alignments may support the existence of a fusion. **(B)** Clinker output of the *KMT2A-MLLT3* gene fusion, visualized in IGV and **(C)** the GViz visualization. The tracks in the Clinker GViz visualization are (top to bottom): a superTranscript scale axis, a read coverage track, a gene boundary track, a protein domain track, a transcript (with exons annotation) track, and a sashimi plot that indicates the fusion breakpoints (dark purple). The breakpoints are also indicated by the vertical lines. In addition to the Clinker tracks, the IGV visualization includes a read support track.

Most fusion calling algorithms use short-read RNA-seq data to report genes involved in potential fusion events as well as the number of reads detected that support these events. Figure2 demonstrates the visualization of a *KMT2A-MLLT3* fusion gene that was detected in a B-cell ALL using JAFFA [16] with and without using Clinker. Visualizing this fusion using IGV without Clinker is done using a split-screen display of the regions of the genome spanning the fusion breakpoints (Fig.2A). While read pairs that span across the fusion breakpoints are viewable (green reads), the transcript context is difficult to discern. In contrast, use of the Clinker superTranscript reference and outputs allows a neater and more informative visualization in IGV that can also display sashimi plots for the fusion support (Fig.2B). Finally, Clinker also outputs a PDF image of the fusion that can be customized (Fig.2C).

### Identification of novel fusion isoforms in *P2RY8-CRLF2*

In order to demonstrate the utility of Clinker to provide visualization and insight into fusion genes, we applied Clinker to six B-cell ALL (B-ALL) samples that carried the *P2RY8-CRLF2* fusion. This fusion gene is reported to be present in ∼7% of B-ALL cases and results in the overexpression of *CRLF2* [20]. The canonical fusion joins the first noncoding (UTR) exon of *P2RY8* to the start of the coding region of *CRLF2* [20]. Commonly, this fusion arises as a result of an interstitial deletion in the Par1 region of chrX or chrY [20]. Interestingly, JAFFA called multiple breakpoints in *CRLF2* for this fusion gene in each of the sequenced B-ALL samples, suggesting different isoforms of this fusion. JAFFA identified the canonical breakpoint in all samples. In addition, each of the six samples also expressed an isoform of *P2RY8-CRLF2* that joined the first exon of *P2RY8* to the 5′ UTR of *CRLF2* and resulted in an in-frame transcript. This alternate fusion also featured the typical GT/AG donor/acceptor motif that exists at the majority of splice junctions [21]. The presence of the alternate fusion isoforms in the samples was confirmed using Reverse Transcription PCR and Sanger sequencing.

**Figure 3.**
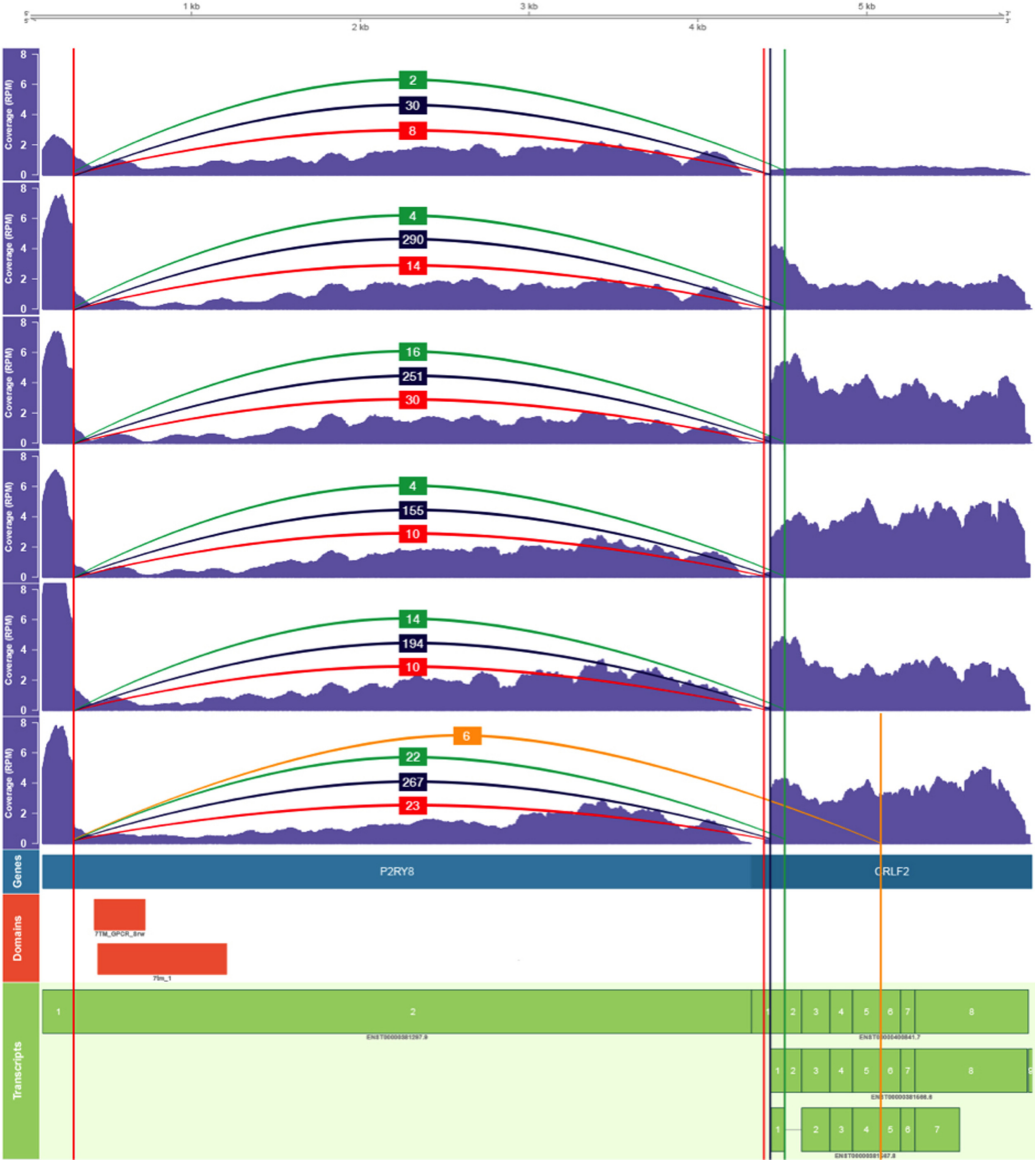
Visualization of six samples containing the *P2RY8-CRLF2* fusion. We combined the Clinker output (mapped reads, fusion superTranscript, and annotation track) for the six samples using Gviz in R. From top to bottom: six coverage tracks with annotated breakpoints demonstrating read support, gene track, protein domains, and gene transcripts. Each sample contains the canonical transcript (navy vertical line) as well as a novel upstream splicing occurring within the 5′UTR exon of*CRLF2* (annotated with the red vertical line over the *CRLF2* gene) along with two other transcripts that are not in-frame. The read support for this Clinker output can be compared to that of JAFFA's in Supplementary Table S1.

We used Clinker to visualize the *P2RY8-CRLF2* fusions in all six samples (Fig. 3). Clinker detected the canonical breakpoint with the highest coverage in all samples (blue lines) and the novel 5′ splice site in all samples at much lower levels (red lines). Interestingly, all six samples had additional splice sites between exon 1 and exon 2 of *CRLF2* (green lines), and one sample had a fourth isoform with a splicing breakpoint between exon 5 and exon 6 of *CRLF2* (yellow lines). However, these additional transcripts are not predicted to be in-frame.

To determine if the alternative, low-abundance, in-frame transcript could drive *CRLF2* overexpression and, in turn, potentially contribute to the biology of ALL driven by *P2RY8-CRLF2* fusions, we cloned the canonical and alternative version of the *P2RY8-CRLF2* fusion, as well as a frameshift version to act as a negative control, into retroviral vectors. The erythroleukemia cell lines (BaF3 cells) were then transduced with these retroviruses to produce cell lines constitutively expressing the in-frame fusions or the negative (frameshift) *P2RY8-CRLF2* control. We measured CRLF2 expression using an anti-human CRLF2 antibody and flow cytometry (Supplementary Fig. S5). The data show that both the canonical fusion and the alternate in-frame transcript can drive *CRLF2* overexpression in BaF3 cells, but that the shorter frame shift transcript does not. These data suggest that alternate transcript isoforms can contribute to the overexpression of CRLF2 in B-ALL.

## Discussion

Here, we present Clinker, a visualization tool for exploring and plotting fusion genes discovered in RNA-seq data. Clinker uses the idea of superTranscripts to build a reference for identified fusions, allowing the raw reads involved in the discovery of fusion genes to be viewed and inspected in IGV. Mapping reads back to this generated fusion gene reference generally results in greater read support for true fusion events. In addition, Clinker annotates transcripts and protein domains, providing far greater insight into the expression levels and structures of the transcripts that make up the fusion gene. Publication-quality figures can be easily generated and refined using R functions. Applying Clinker to real data demonstrated that alternative splicing could be detected within a single fusion gene. Our examination of the *P2RY8-CRLF2* fusions indicates that these alternate isoforms exist in the primary samples and may have biological relevance, as it appears capable of encoding a functional CRLF2 protein.

## Availability of source code and requirements

- Project name: Clinker
- Project home page: https://github.com/Oshlack/Clinker/
- Operating systems: 64 bit Linux or Mac OS X
- Programming language: Python, R, Bash
- Other requirements: STAR, Samtools and Gviz
- License: MIT
- RRID: Clinker, RRID:SCR_016140

## Availability of supporting data

Snapshots of the code are available from the *GigaScience* GigaDB repository [22].

## Abbreviations

ALL: acute lymphoblastic leukemia; PCR: polymerase chain reaction; RNA-seq: RNA sequencing.

## Competing interests

The authors declare that they have no competing interests

## Ethics

The project was approved by the Human Research Ethics Committee of the Royal Children's Hospital (RCH) ( 34127D). All samples were obtained from the Children's Cancer Centre Tissue Bank, which is managed under the ethics governance approved by the Human Research Ethics Committee of the RCH (33207K)

## Funding

This work was supported by grants from the Australian National Health and Medical Research Council (project grants to A.O. [1140626], I.J.M. [1145912], Career Development Fellowship to A.O. [1126157] and Independent Research Institutes Infrastructure Support Scheme grant [9000220]), the Cancer Council Victoria (grant-in-aid to I.J.M. [1124178]), a Victorian State Government Operational Infrastructure Support grant; a Victorian Cancer Agency fellowship (to I.J.M.), and a Felton Bequest (to I.J.M.). P.G.E. acknowledges the funding support of the Children's Cancer Foundation.

## Author contributions

B.M.S. wrote the software, performed analysis, and co-wrote original draft. N.M.D. provided conceptualization, supervision, reviewing, and editing. A.D.K.H. contributed to initial conceptualization and analysis. R.B. performed investigations and validation and reviewed and edited the manuscript. I.J.M. and P.G.E. contributed to supervision, resources, funding acquisition, and reviewing and editing. In addition, P.G.E. performed lab-based investigations and validations. A.O. wrote and edited the manuscript, performed supervision, and contributed to conceptualization, funding acquisition, and methodology.

## Supplementary Material

GIGA-D-18-00019_Original_Submission.pdf

GIGA-D-18-00019_Revision_1.pdf

GIGA-D-18-00019_Revision_2.pdf

Response_to_Reviewer_Comments_Original_Submission.pdf

Response_to_Reviewer_Comments_Revision_1.pdf

Reviewer_1_Report_(Original_Submission) -- Brian Haas 2/2/2018 Reviewed

Reviewer_2_Report_(Original_Submission) -- Andreas Hoff 2/6/2018 Reviewed

Reviewer_1_Report_(Revision_1) -- Andreas Hoff 5/24/2018 Reviewed

Supplemental Files
