# Supplementary material for: Clinker: visualizing fusion genes detected in RNA-seq data
Source: Gigascience. 2018 Jul 4;7(7):giy079. doi: 10.1093/gigascience/giy079 (PMC6065480; doi:10.1093/gigascience/giy079)
Supplement: Supplemental Files [file giy079_supplemental_files.docx]

Clinker: visualising fusion genes
detected in RNA-seq data.
Supplementary Figures and Tables.

Breon M Schmidt, Nadia M Davidson, Anthony DK Hawkins,
Ray Bartolo, Ian J Majewski, Paul G Ekert, Alicia Oshlack

_____________

**Clinker Read Support


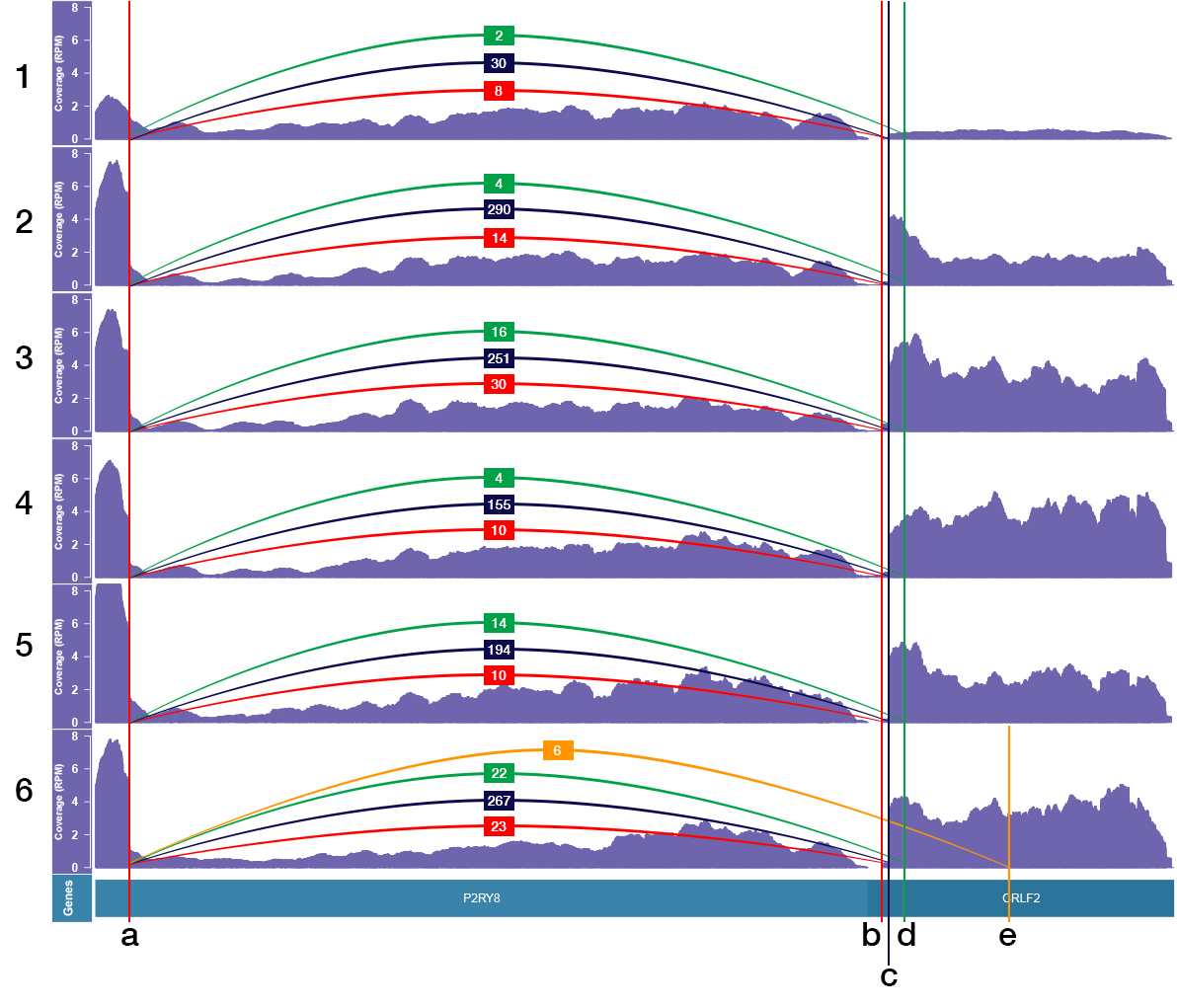

Figure S1.** Modified clinker output that demonstrates the coverage and breakpoints of six samples containing the P2RY8-CRLF2 fusion gene.
 **JAFFA and Clinker spanning read support for the P2RY8-CRLF2 fusions found in the samples above**

| **Sample** | **Fusion a-b** | | **Fusion a-c** | | **Fusion a-d** | | **Fusion a-e** | |
| --- | --- | --- | --- | --- | --- | --- | --- | --- |
|  | **JAFFA** | **Clinker** | **JAFFA** | **Clinker** | **JAFFA** | **Clinker** | **JAFFA** | **Clinker** |
| 1 | N/A | 14 | 53 | 290 | 2 | 4 | N/A | N/A |
| 2 | N/A | 10 | 55 | 155 | 4 | 4 | N/A | N/A |
| 3 | 5 | 10 | 36 | 194 | 10 | 14 | N/A | N/A |
| 4 | 6 | 23 | 57 | 267 | 10 | 22 | N/A | 6 |
| 5 | 2 | 8 | 10 | 30 | 3 | 2 | N/A | N/A |
| 6 | 4 | 30 | 51 | 251 | 8 | 16 | N/A | N/A |

**Table S1.** Number of spanning reads (reads that split across the breakpoint) discovered by JAFFA (JAFFA filters out breakpoints that are not inframe) and Clinker.

Table S1 indicates that Clinker alignment produces more read support from spanning reads. This is intuitive given that Clinker concatenates the superTranscripts from both genes to form a single reference sequence, which the STAR aligner would more efficiently align to.

# Spurious Read Alignments
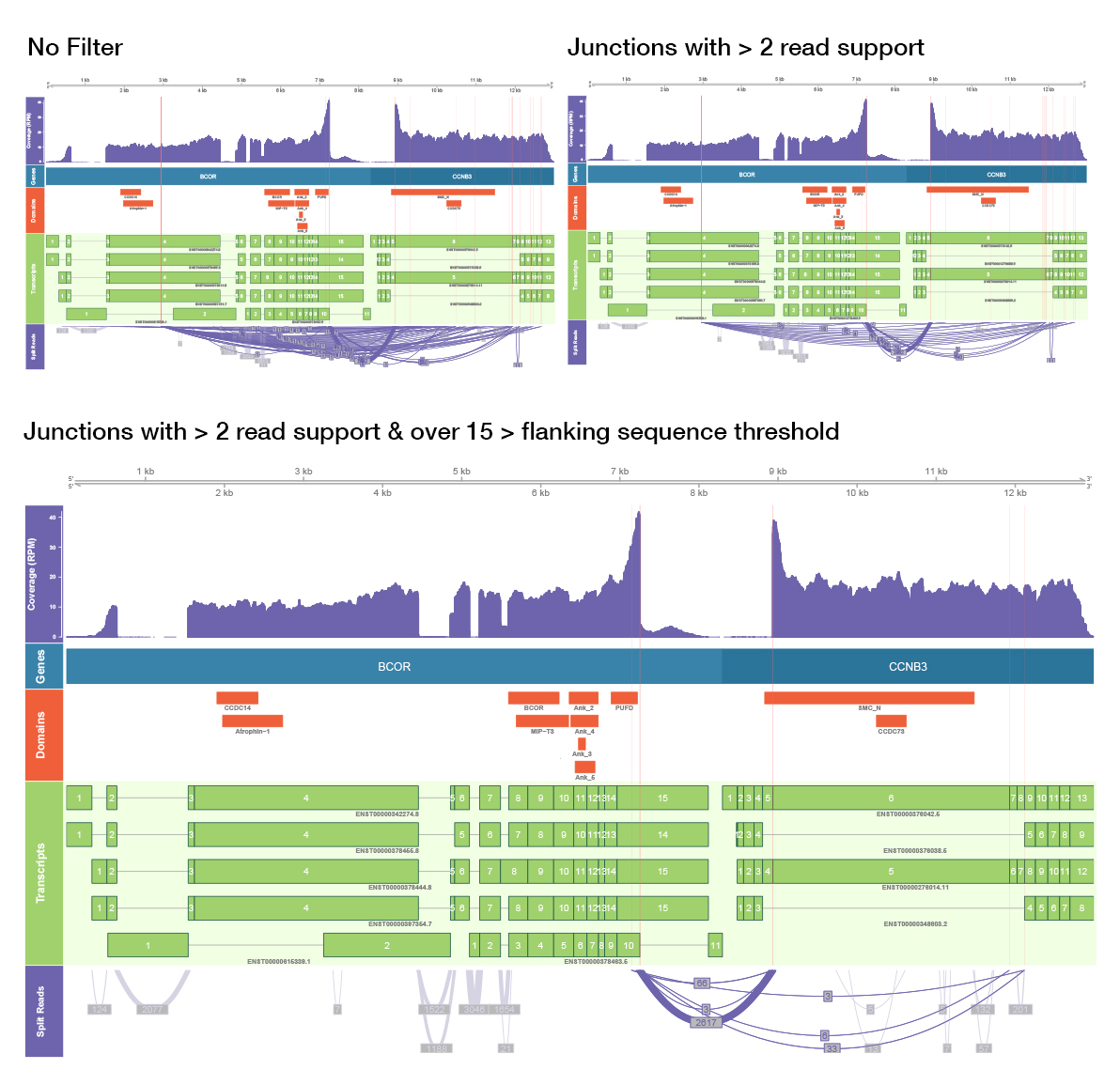
 Figure S2. There is substantial reduction of noisy alignments when minimum flanking sequence and minimum junction support thresholds are defined. Minimum flanking sequence is increased to 15 in this case due the large number of split read alignments.

Short CAAGGTTGCTGGACAGATGGAACTGGAAGGGCAGCCGTCTGCCGCCCACGAACACCTTCT 60

Canon CAAGGTTGCTGGACAGATGGAACTGGAAGGGCAGCCGTCTGCCGCCCACGAACACCTTCT 60

FL CAAGGTTGCTGGACAGATGGAACTGGAAGGGCAGCCGTCTGCCGCCCACGAACACCTTCT 60

************************************************************


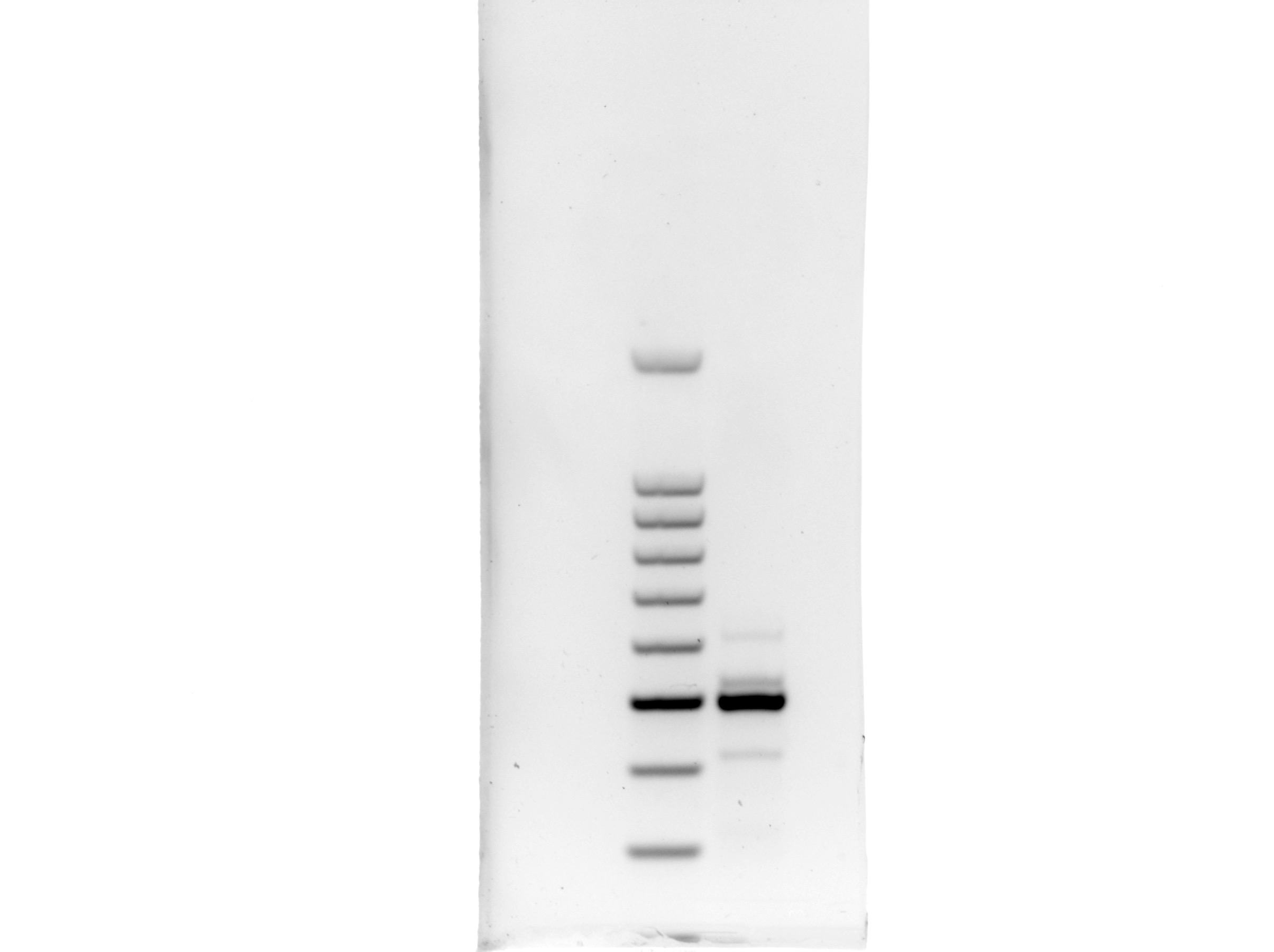


600bp

500bp

400bp

Short CAAGCACTTTGAGTGACCACGGCTTGCAAGCTGGTGGCTGGCCCCCCGAGTCCCGGGCTC 120

Canon CAAGCACTTTGAGTGACCACGGCTTGCAAGCTGGTGGCTGGCCCCCCGAGTCCCGGGCTC 120

FL CAAGCACTTTGAGTGACCACGGCTTGCAAGCTGGTGGCTGGCCCCCCGAGTCCCGGGCTC 120

************************************************************

Short TGAGGCACGGCCGTCGACTTAAGCGTTGCATCCTGTTACCTGGAGACCCTCTGAGCTCTC 180

Canon TGAGGCACGGCCGTCGACTTAAGCGTTGCATCCTGTTACCTGGAGACCCTCTGAGCTCTC 180

FL TGAGGCACGGCCGTCGACTTAAGCGTTGCATCCTGTTACCTGGAGACCCTCTGAGCTCTC 180

************************************************************

Short ACCTGCTACTTCTGCCGCTGCTTCTGCAC------------------------------- 209

Canon ACCTGCTACTTCTGCCGCTGCTTCTGCACAG----------------------------- 211

FL ACCTGCTACTTCTGCCGCTGCTTCTGCACAGGTCCCTGAGGACCTCACTCTCCACTCCTG 240

*****************************

Short ------------------------------------------------------------ 209

Canon ------GCATGGGGCGGCTGGTTCTGCTGTGGGGAGCTGCCGTCTTTCTGCTGGGAGGCT 265

FL TTTCAGGCATGGGGCGGCTGGTTCTGCTGTGGGGAGCTGCCGTCTTTCTGCTGGGAGGCT 300

Short ----------------------------AGAAGGAGTACAGATTCAGATCATCTACTTCA 241

Canon GGATGGCTTTGGGGCAAGGAGGAGCAGCAGAAGGAGTACAGATTCAGATCATCTACTTCA 325

FL GGATGGCTTTGGGGCAAGGAGGAGCAGCAGAAGGAGTACAGATTCAGATCATCTACTTCA 360

********************************

Short ATTTAGAAACCGTGCAGGTGACATGGGATGCCAGCAAATACTCCAGGACCAACCTGACTT 301

Canon ATTTAGAAACCGTGCAGGTGACATGGAATGCCAGCAAATACTCCAGGACCAACCTGACTT 385

FL ATTTAGAAACCGTGCTGGTGACATGGAATGCCAGCAAATACTCCAGGACCAACCTGACTT 420

*************** ********** *********************************

Short TCCACTACAGATTCAACGGTGATGAGGCCTATGACCAGTGTACCAACTACCTTCTCCAGG 361

Canon TCCACTACAGATTCAACGGTGATGAGGCCTATGACCAGTGCACCAACTACCTTCTCCAGG 445

FL TCCACTACAGATTCAACGGTGATGAGGCCTATGACCAGTGCACCAACTACCTTCTCCAGG 480

**************************************** *******************

Short AAGGTCACACTTCGGGGTGCCTCCTAGACGCAGAGCAGCGAGACGACATTCTCTATT 418

Canon AAGGTCACACTTCGGGGTGCCTCCTAGACGCAGAGCAGCGAGACGACATTCTCTATT 502

FL AAGGTCACACTTCGGGGTGCCTCCTAGACGCAGAGCAGCGAGACGACATTCTCTATT 537

*********************************************************

**Figure S3**. DNA gel showing cDNAs amplified from patient 6 (see S1 for clinker output) by RT-PCR using primers that flank the P2RY8-CRLF2 sequence identified by RNAseq. Arrows represent the various transcripts; Red = FL (full length fusion), Blue = Canon (canonical fusion) and Green = Short. The corresponding Sanger sequencing results are also shown with primer sequences used for PCR underlined. The CRLF2 start site (ATG) is highlighted in Green. The CRLF2 start site is not present in the Short transcript and is therefore not predicted to be in frame or to produce CRLF2 protein.

**Figure S4.** Sanger sequencing results of P2RY8-CRLF2 cDNAs cloned from patient 6 (see S1 for clinker output). These cDNAs were cloned into a pMSCV-GFP retroviral expression vector and transduced into BaF3 cells. Underlined sequences show the sites where PCR primer align, while the highlighted sequences show P2RY8 (green) and CRLF2 (purple) transcription start sites, CRLF2 5’ UTR (blue) and the CRLF2 stop codon (red).

Canon ------------------------------------------------------------ 0

FrameShift CCCTGCACATGAGTGTTCAGACAGTTACAGAGGAAATGTCACAACACACTTCCTTTCCAC 60

Alternate CCCTGCACATGAGTGTTCAGACAGTTACAGAGGAAATGTCACAACACACTTCCTTTCCAC 60

Canon ------------------------------------------------------------ 0

FrameShift CTAAGCCTGAGTCGCAACCGTGGTGGTGCGCTGGGGGGTGGAGTTTGCAGAATTTGCATT 120

Alternate CTAAGCCTGAGTCGCAACCGTGGTGGTGCGCTGGGAGGTGGAGTTTGCAGAATTTGCATT 120

Canon --------------------------------------GCGGCCGCCTTTGCAAGGTTGC 22

FrameShift CGGAGACAGTCGTGCCAGCCGGTGGGCCACCCAGCGAAGCGGCCGCCTTTGCAAGGTTGC 180

Alternate CGGAGACAGTCGTGCCAGCCGGTGGGCCACCCAGCGAAGCGGCCGCCTTTGCAAGGTTGC 180

**********************

Canon TGGACAGATGGAACTGGAAGGGCAGCCGTCTGCCGCCCACGAACACCTTCTCAAGCACTT 82

Alternate TGGACAGATGGAACTGGAAGGGCAGCCGTCTGCCGCCCACGAACACCTTCTCAAGCACTT 240

FrameShift TGGACAGATGGAACTGGAAGGGCAGCCGTCTGCCGCCCACGAACACCTTCTCAAGCACTT 240

************************************************************

Canon TGAGTGACCACGGCTTGCAAGCTGGTGGCTGGCCCCCCGAGTCCCGGGCTCTGAGGCACG 142

FrameShift TGAGTGACCACGGCTTGCAAGCTGGTGGCTGGCCCCCCGAGTCCCGGGCTCTGAGGCACG 300

Alternate TGAGTGACCACGGCTTGCAAGCTGGTGGCTGGCCCCCCGAGTCCCGGGCTCTGAGGCACG 300

************************************************************

Canon GCCGTCGACTTAAGCGTTGCATCCTGTTACCTGGAGACCCTCTGAGCTCTCACCTGCTAC 202

FrameShift GCCGTCGACTTAAGCGTTGCATCCTGTTACCTGGAGACCCTCTGAGCTCTCACCTGCTAC 360

Alternate GCCGTCGACTTAAGCGTTGCATCCTGTTACCTGGAGACCCTCTGAGCTCTCACCTGCTAC 360

************************************************************

Canon TTCTGCCGCTGCTTCTGCACAG-----------------------------------GCA 227

FrameShift TTCTGCCGCTGCTTCTGCACAG-----------------------------------GCA 385

Alternate TTCTGCCGCTGCTTCTGCACAGGTCCCTGAGGACCTCACTCTCCACTCCTGTTTCAGGCA 420

********************** ***

Canon TGGGGCGGCTGGTTCTGCTGTGGGGAGCTGCCGTCTTTCTGCTGGGAGGCTGGATGGCTT 287

FrameShift TGGGGCGGCTGGTTCTGCTGTGGGGAGCTGCCGTCTTTCTGCTGGGAGGCTGGATGGCTT 445

Alternate TGGGGCGGCTGGTTCTGCTGTGGGGAGCTGCCGTCTTTCTGCTGGGAGGCTGGATGGCTT 480

************************************************************

Canon TGGGGCAAGGAGGAGCAGCAGAAGGAGTACAGATTCAGATCATCTACTTCAATTTAGAAA 347

FrameShift TGGGGCAAGGAGGAGCAGCAGAAGGAGTACAGATTCAGATCATCTACTTCAATTTAGAAA 505

Alternate TGGGGCAAGGAGGAGCAGCAGAAGGAGTACAGATTCAGATCATCTACTTCAATTTAGAAA 540

************************************************************

Canon CCGTGCAGGTGACATGGAATGCCAGCAAATACTCCAGGACCAACCTGACTTTCCACTACA 407

FrameShift CCGTGCAGGTGACATGGAATGCCAGCAAATACTCCAGGACCAACCTGACTTTCCACTACA 565

Alternate CCGTGCAGGTGACATGGAATGCCAGCAAATACTCCAGGACCAACCTGACTTTCCACTACA 600

************************************************************

Canon GATTCAACGGTGATGAGGCCTATGACCAGTGCACCAACTACCTTCTCCAGGAAGGTCACA 467

FrameShift GATTCAACGGTGATGAGGCCTATGACCAGTGCACCAACTACCTTCTCCAGGAAGGTCACA 625

Alternate GATTCAACGGTGATGAGGCCTATGACCAGTGCACCAACTACCTTCTCCAGGAAGGTCACA 660

************************************************************

Canon CTTCGGGGTGCCTCCTAGACGCAGAGCAGCGAGACGACATTCTCTATTTCTCCATCAGGA 527

FrameShift CTTCGGGGTGCCTCCTAGACGCAGAGCAGCGAGACGACATTCTCTATTTCTCCATCAGGA 685

Alternate CTTCGGGGTGCCTCCTAGACGCAGAGCAGCGAGACGACATTCTCTATTTCTCCATCAGGA 720

************************************************************

Canon ATGGGACGCACCCCGTTTTCACCGCAAGTCGCTGGATGGTTTATTACCTGAAACCCAGTT 587

FrameShift ATGGGACGCACCCCGTTTTCACCGCAAGTCGCTGGATGGTTTATTACCTGAAACCCAGTT 745

Alternate ATGGGACGCACCCCGTTTTCACCGCAAGTCGCTGGATGGTTTATTACCTGAAACCCAGTT 780

************************************************************

Canon CCCCGAAGCACGTGAGATTTTCGTGGCATCAGGATGCAGTGACGGTGACGTGTTCTGACC 647

FrameShift CCCCGAAGCACGTGAGATTTTCGTGGCATCAGGATGCAGTGACGGTGACGTGTTCTGACC 805

Alternate CCCCGAAGCACGTGAGATTTTCGTGGCATCAGGATGCAGTGACGGTGACGTGTTCTGACC 840

************************************************************

Canon TGTCCTACGGGGATCTCCTCTATGAGGTTCAGTACCGGAGCCCCTTCGACACCGAGTGGC 707

FrameShift TGTCCTACGGGGATCTCCTCTATGAGGTTCAGTACCGGAGCCCCTTCGACACCGAGTGGC 865

Alternate TGTCCTACGGGGATCTCCTCTATGAGGTTCAGTACCGGAGCCCCTTCGACACCGAGTGGC 900

************************************************************

Canon AGTCCAAACAGGAAAATACCTGCAACGTCACCATAGAAGGCTTGGATGCCGAGAAGTGTT 767

FrameShift AGTCCAAACAGGAAAATACCTGCAACGTCACCATAGAAGGCTTGGATGCCGAGAAGTGTT 925

Alternate AGTCCAAACAGGAAAATACCTGCAACGTCACCATAGAAGGCTTGGATGCCGAGAAGTGTT 960

************************************************************

Canon ACTCTTTCTGGGTCAGGGTGAAGGCTATGGAGGATGTATATGGGCCAGACACATACCCAA 827

FrameShift ACTCTTTCTGGGTCAGGGTGAAGGCTATGGAGGATGTATATGGGCCAGACACATACCCAA 985

Alternate ACTCTTTCTGGGTCAGGGTGAAGGCTATGGAGGATGTATATGGGCCAGACACATACCCAA 1020

************************************************************

Canon GCGACTGGTCAGAGGTGACATGCTGGCAGAGAGGCGAGATTCGGGATGCCTGTGCAGAGA 887

FrameShift GCGACTGGTCAGAGGTGACATGCTGGCAGAGAGGCGAGATTCGGGATGCCTGTGCAGAGA 1045

Alternate GCGACTGGTCAGAGGTGACATGCTGGCAGAGAGGCGAGATTCGGGATGCCTGTGCAGAGA 1080

************************************************************

Canon CACCAACGCCTCCCAAACCAAAGCTGTCCAAATTTATTTTAATTTCCAGCCTGGCCATCC 947

FrameShift CACCAACGCCTCCCAAACCAAAGCTGTCCAAATTTATTTTAATTTCCAGCCTGGCCATCC 1105

Alternate CACCAACGCCTCCCAAACCAAAGCTGTCCAAATTTATTTTAATTTCCAGCCTGGCCATCC 1140

************************************************************

Canon TTCTGATGGTGTCTCTCCTCCTTCTGTCTTTATGGAAATTATGGAGAGTGAGGAAGTTTC 1007

FrameShift TTCTGATGGTGTCTCTCCTCCTTCTGTCTTTATGGAAATTATGGAGAGTGAGGAAGTTTC 1165

Alternate TTCTGATGGTGTCTCTCCTCCTTCTGTCTTTATGGAAATTATGGAGAGTGAGGAAGTTTC 1200

************************************************************

Canon TCATTCCCAGCGTGCCAGACCCGAAATCCATCTTCCCCGGGCTCTTTGAGATACACCAAG 1067

FrameShift TCATTCCCAGCGTGCCAGACCCGAAATCCATCTTCCCCGGGCTCTTTGAGATACACCAAG 1225

Alternate TCATTCCCAGCGTGCCAGACCCGAAATCCATCTTCCCCGGGCTCTTTGAGATACACCAAG 1260

************************************************************

Canon GGAACTTCCAGGAGTGGATCACAGACACCCAGAACGTGGCCCACCTCCACAAGATGGCAG 1127

FrameShift GGAACTTCCAGGAGTGGATCACAGACACCCAGAACGTGGCCCACCTCCACAAGATGGCAG 1285

Alternate GGAACTTCCAGGAGTGGATCACAGACACCCAGAACGTGGCCCACCTCCACAAGATGGCAG 1320

************************************************************

Canon GTGCAGAGCAAGAAAGTGGCCCCGAGGAGCCCCTGGTAGTCCAGTTGGCCAAGACTGAAG 1187

FrameShift GTGCAGAGCAAGAAAGTGGCCCCGAGGAGCCCCTGGTAGTCCAGTTGGCCAAGACTGAAG 1345

Alternate GTGCAGAGCAAGAAAGTGGCCCCGAGGAGCCCCTGGTAGTCCAGTTGGCCAAGACTGAAG 1380

************************************************************

Canon CCGAGTCTCCCAGGATGCTGGACCCACAGACCGAGGAGAAAGAGGCCTCTGGGGGATCCC 1247

FrameShift CCGAGTCTCCCAGGATGCTGGACCCACAGACCGAGGAGAAAGAGGCCTCTGGGGGATCCC 1405

Alternate CCGAGTCTCCCAGGATGCTGGACCCACAGACCGAGGAGAAAGAGGCCTCTGGGGGATCCC 1440

************************************************************

Canon TCCAGCTTCCCCACCAGCCCCTCCAAGGTGGTGATGTGGTCACAATCGGGGGCTTCACCT 1307

FrameShift TCCAGCTTCCCCACCAGCCCCTCCAAGGTGGTGATGTGGTCACAATCGGGGGCTTCACCT 1465

Alternate TCCAGCTTCCCCACCAGCCCCTCCAAGGTGGTGATGTGGTCACAATCGGGGGCTTCACCT 1500

************************************************************

Canon TTGTGATGAATGACCGCTCCTACGTGGCGTTGTGA 1342

FrameShift TTGTGATGAATGACCGCTCCTACGTGGCGTTGTGA 1500

Alternate TTGTGATGAATGACCGCTCCTACGTGGCGTTGTGA 1535

***********************************


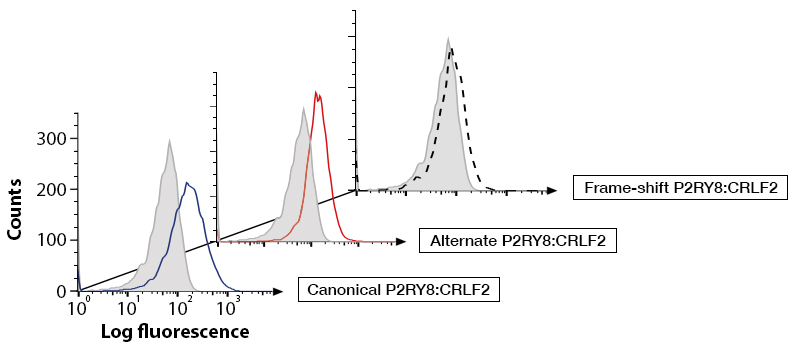


**Figure S5.** Representative histograms of CRLF2 expression in BaF3 cells measured by Flow Cytometry. BaF3 cells were transfected with either the canonical *P2RY8-CRLF2* fusion, the alternate in-frame fusion that includes the 1st exon of *P2RY8* and the 5’UTR of *CRLF2* or a third isoform detected in patient samples that results in a frame shift and a premature stop codon. The blue, red and dashed histograms show CRLF2 expression detected using an anti-CRLF2 antibody. The gray shaded histogram shows unstained controls. The same unstained control histogram is shown in each case as this data is from a single representative experiment.
