# Supplementary material for: Clinker: visualizing fusion genes detected in RNA-seq data
Source: Gigascience. 2018 Jul 4;7(7):giy079. doi: 10.1093/gigascience/giy079 (PMC6065480; doi:10.1093/gigascience/giy079)
Supplement: Response_to_Reviewer_Comments_Original_Submission.pdf [file giy079_response_to_reviewer_comments_original_submission.pdf]

Author's Response To Reviewer Comments

Close

Reviewer reports:

Reviewer #1: The Clinker software provides a useful tool for visualizing the evidence supporting fusion transcript predictions, and it further builds on the authors' earlier work involving superTranscripts (essentially intron-free, concatenated exonic regions representing the intronless gene sequence, aiming to simplify analyses in multiple contexts). Given a list of candidate fusions provided as input, Clinker constructs a fusion-superTranscript, and uses STAR to align reads to it along with the remaining set of superTranscripts reflecting the rest of the transcriptome. In doing so, Clinker identifies the evidence supporting the fusion event(s) between the candidate fusion genes. The data are readily navigated in the IGV viewer, and Clinker includes additional reporting that generates static plots in pdf format, which showcase the evidence supporting the fusion events in publication-quality images.
The role of Clinker is very similar to that of our own
FusionInspector tool (not yet published,
https://github.com/FusionInspector/FusionInspector/wiki), as referenced by the authors, where FusionInspector 'shrinks' introns as opposed to creating superTranscripts, and searches the entire genome as opposed to just the exonic sequences. These methods are quite useful and expand on the abilities of researchers to explore the evidence supporting fusion predictions, particularly useful for exploring novel fusion events and isoforms that may be clinically relevant. The authors demonstrate this nicely with Clinker by exploring novel breakpoints of fusions in acute lymphoblastic leukemia.

I was able to download and run the software, hitting a minor hiccup where I needed to make the 'Clinker/plotit/fst_plot_prep.sh' script executable and then rerun the pipeline. The documentation on the software wiki including the vignette was quite helpful, but was also clearly in an unfinished state and deserves more attention prior to publication.

We have now fixed the issue with the 'Clinker/plotit/fst_plot_prep.sh' script and made some small edits and additions to the vignette. We believe it contains as much background as needed and we will link to the paper once it is published.

Reviewer #2: This manuscript describes the implementation of "Clinker", a bioinformatic tool designed to visualize fusion transcripts detected in RNA-sequencing data. The tool applies a previously developed (by the authors) superTranscript method to generate a collapsed reference, with a single sequence representing the union of all transcript variants for a single gene. The method is here modified to also generate a fusion-superTranscript reference for each fusion as input from the results of a fusion finder software. The authors have conveniently designed the approach to only take chromosome and breakpoint information from the fusion finder, information that most of the many fusion finders available will provide. Further, the splice aware aligner STAR is used to align raw RNA-seq reads back to the newly generated superTranscript reference. The method also implements visualization with the Gviz R package and generates informative figures of the fusion genes of interest.
Also, a reference file, annotations and aligned reads are output that can conveniently be visualized using IGV. This provides additional usability compared to visualizing RNA-seq reads aligned to the standard genome reference with IGV. The manuscript is clearly written

and the Github page https://github.com/Oshlack/Clinker is well organized with clear instructions and examples for using Clinker. The code is also well formatted and easily viewable, although I have not had the time to go into all of it in too much detail.

I have the following comments:

1. My main comment to the manuscript and the method is that I do not think that Clinker and the manuscript fulfills the authors claim that it is a tool that provides "an appreciation of their complexity that is not available using other methods" (line 13,14: page 2). Specifically, our " " tool is able to generate a more variable array of plots to investigate a specific fusion transcript or the fusion transcript landscape in a given sample. Also, using the fusion transcript plot function, it is possible to generate a collapsed representation of all exons that potentially are a part of a specific fusion transcript. However, I do agree that Clinker adds to the relatively small repertoire of tools that can be used for the daunting task of appreciating the complexity of fusion transcripts. Of special importance is the ability to show supporting reads of alternative fusion variants, not necessarily reported by the fusion finder. The authors use this feature to nicely show
the presence of novel breakpoints within the P2RY8-CRLF2 fusion gene in B-ALL.

We have reworded the manuscript especially the introduction to make it clear that Clinker is not the only tool designed to visualize fusion genes.

2. The authors write that they have confirmed the presence of the P2RY8-CRLF2 fusion isoforms with PCR and Sanger sequencing. However, I do not see this supporting data. Additionally, the authors have performed functional experiments and find that a novel fusion isoform of P2RY8-CRLF2 drive CRLF3 overexpression in BaF3 cells. However, I am missing the supporting data behind these conclusions. In addition to the resulting flow cytometry data, I would have liked to see a sequence confirmation of the cloned canonical and alternative variants of P2RY8-CRLF2 and a validation of the resulting constitutive expression of these isoforms in transduced BaF3 cells.

We have now added the supporting sanger sequencing data for three alternative break points of the P2RY8-CRLF2 fusion into the supplementary file. We used flow cytometry to confirm that it is only the fusion transcripts that retain the canonical ATG initiation codon that produce detectable CRLF2 protein in transfected BaF3 cells.

3. Some suggestions to the tool: For the plots output by Gviz, could the transcripts originating from each gene be colored in separate colors to distinguish the different parts of the fusion transcript? I also miss some information of the original genomic location of the partner genes, for instance the chromosome and start/stop coordinates.

Based on these suggestions we have now coloured the transcripts from each gene in different colours and included the genomic coordinates.

4. I cannot see a reference or accession number to where (or if) the RNA-seq data from the six B-cell ALL patient samples used in the manuscript have been deposited. According to GigaScience's standards for data sharing, this should be included and/or excerpts of the data could be used as example data for the tool.

We have now included a reference for our GEO data.

Minor comments:

- The quality of the figures in the manuscript is somewhat poor and should be improved. Especially for figure 2, the resolution and the size of the IGV screenshots should be increased.

This has been corrected

- Nomenclature: When referring to the P2RY8-CRLF2 fusion, there is a mix of using italics and regular throughout the manuscript. Italics should be used when referring to the fusion on a gene level and regular font when referring to proteins (e.g. line 15: page 9)

We have addressed the inconsistency.

- A reference to the original discovery of the BCR-ABL1 oncogene should be added (line 31,32: page 1).

The reference has been added.

I might be missing this, but from the manuscript and the Github wiki, I can't find an option to run Clinker to visualize all nominated fusion transcripts in an input file. Only the option to input each fusion name in the "fusions" parameter. In the manuscript, under software requirements, a benchmarking of running 2007 fusion genes is mentioned. I think this is of importance, as this is a nice feature of Clinker; to be able to visualize many fusion genes rapidly, for exploration of fusion gene predictions that are often hampered by false positives.

All fusion in the input file are actually run through Clinker automatically regardless of whether you use the “fusion” parameter or not. You can then look through every one in the same IGV instance. We have tried to make this clearer in the paper.

In the Github wiki, the parameter to assign which columns contains chromosome and coordinate of breakpoints is sometime referred to as "pos" and sometimes as "col". It seems to me that only "col" works as I get an error when running with "pos".

We have now fixed this.

Close
