# Supplementary material for: Clinker: visualizing fusion genes detected in RNA-seq data
Source: Gigascience. 2018 Jul 4;7(7):giy079. doi: 10.1093/gigascience/giy079 (PMC6065480; doi:10.1093/gigascience/giy079)
Supplement: Response_to_Reviewer_Comments_Revision_1.pdf [file giy079_response_to_reviewer_comments_revision_1.pdf]

Author's Response To Reviewer Comments

Close

We have now made the formatting changes requested

Close
