# Supplementary material for: Clinker: visualizing fusion genes detected in RNA-seq data
Source: Gigascience. 2018 Jul 4;7(7):giy079. doi: 10.1093/gigascience/giy079 (PMC6065480; doi:10.1093/gigascience/giy079)
Supplement: Reviewer_1_Report_(Original_Submission) -- Brian Haas — 2/2/2018 Reviewed [file giy079_reviewer_1_report_(original_submission).pdf]

**Reviewer Report**

**Title: Clinker: visualising fusion genes detected in RNA-seq data**

**Version: Original Submission** **Date:** 2/2/2018

**Reviewer name: Brian Haas**

**Reviewer Comments to Author:**

he Clinker software provides a useful tool for visualizing the evidence supporting fusion transcript predictions, and it further builds on the authors' earlier work involving superTranscripts (essentially intron-free, concatenated exonic regions representing the intronless gene sequence, aiming to simplify analyses in multiple contexts). Given a list of candidate fusions provided as input, Clinker constructs a fusion-superTranscript, and uses STAR to align reads to it along with the remaining set of superTranscripts reflecting the rest of the transcriptome. In doing so, Clinker identifies the evidence supporting the fusion event(s) between the candidate fusion genes. The data are readily navigated in the IGV viewer, and Clinker includes additional reporting that generates static plots in pdf format, which showcase the evidence supporting the fusion events in publication-quality images. The role of Clinker is very similar to that of our own FusionInspector tool (not yet published, https://github.com/FusionInspector/FusionInspector/wiki), as referenced by the authors, where FusionInspector 'shrinks' introns as opposed to creating superTranscripts, and searches the entire genome as opposed to just the exonic sequences. These methods are quite useful and expand on the abilities of researchers to explore the evidence supporting fusion predictions, particularly useful for exploring novel fusion events and isoforms that may be clinically relevant. The authors demonstrate this nicely with Clinker by exploring novel breakpoints of fusions in acute lymphoblastic leukemia. I was able to download and run the software, hitting a minor hiccup where I needed to make the 'Clinker/plotit/fst_plot_prep.sh' script executable and then rerun the pipeline. The documentation on the software wiki including the vignette was quite helpful, but was also clearly in an unfinished state and deserves more attention prior to publication.

**Level of Interest**

Please indicate how interesting you found the manuscript: An article of importance in its field

**Quality of Written English**

Please indicate the quality of language in the manuscript: Acceptable

**Declaration of Competing Interests**

Please complete a declaration of competing interests, considering the following questions:

- Have you in the past five years received reimbursements, fees, funding, or salary from an organisation that may in any way gain or lose financially from the publication of this manuscript, either now or in the future?
- Do you hold any stocks or shares in an organisation that may in any way gain or lose financially from the publication of this manuscript, either now or in the future?

- Do you hold or are you currently applying for any patents relating to the content of the manuscript?
- Have you received reimbursements, fees, funding, or salary from an organization that holds or has applied for patents relating to the content of the manuscript?
- Do you have any other financial competing interests?
- Do you have any non-financial competing interests in relation to this paper?

If you can answer no to all of the above, write 'I declare that I have no competing interests' below. If your reply is yes to any, please give details below.

None

I agree to the open peer review policy of the journal. I understand that my name will be included on my report to the authors and, if the manuscript is accepted for publication, my named report including any attachments I upload will be posted on the website along with the authors' responses. I agree for my report to be made available under an Open Access Creative Commons CC-BY license (http://creativecommons.org/licenses/by/4.0/). I understand that any comments which I do not wish to be included in my named report can be included as confidential comments to the editors, which will not be published.

I agree to the open peer review policy of the journal

To further support our reviewers, we have joined with Publons, where you can gain additional credit to further highlight your hard work (see: https://publons.com/journal/530/gigascience). On publication of this paper, your review will be automatically added to Publons, you can then choose whether or not to claim your Publons credit. I understand this statement. Yes
