# Supplementary material for: Clinker: visualizing fusion genes detected in RNA-seq data
Source: Gigascience. 2018 Jul 4;7(7):giy079. doi: 10.1093/gigascience/giy079 (PMC6065480; doi:10.1093/gigascience/giy079)
Supplement: Reviewer_2_Report_(Original_Submission) -- Andreas Hoff — 2/6/2018 Reviewed [file giy079_reviewer_2_report_(original_submission).pdf]

Reviewer Report

Title: Clinker: visualising fusion genes detected in RNA-seq data

Version: Original Submission **Date:** 2/6/2018

Reviewer name: Andreas Hoff

Reviewer Comments to Author:

This manuscript describes the implementation of "Clinker", a bioinformatic tool designed to visualize fusion transcripts detected in RNA-sequencing data. The tool applies a previously developed (by the authors) superTranscript method to generate a collapsed reference, with a single sequence representing the union of all transcript variants for a single gene. The method is here modified to also generate a fusion-superTranscript reference for each fusion as input from the results of a fusion finder software. The authors have conveniently designed the approach to only take chromosome and breakpoint information from the fusion finder, information that most of the many fusion finders available will provide. Further, the splice aware aligner STAR is used to align raw RNA-seq reads back to the newly generated superTranscript reference. The method also implements visualization with the Gviz R package and generates informative figures of the fusion genes of interest. Also, a reference file, annotations and aligned reads are output that can conveniently be visualized using IGV. This provides additional usability compared to visualizing RNA-seq reads aligned to the standard genome reference with IGV. The manuscript is clearly written and the Github page https://github.com/Oshlack/Clinker is well organized with clear instructions and examples for using Clinker. The code is also well formatted and easily viewable, although I have not had the time to go into all of it in too much detail.I have the following comments:1. My main comment to the manuscript and the method is that I do not think that Clinker and the manuscript fulfills the authors claim that it is a tool that provides "an appreciation of their complexity that is not available using other methods" (line 13,14: page 2). Specifically, our "Chimeraviz" tool is able to generate a more variable array of plots to investigate a specific fusion transcript or the fusion transcript landscape in a given sample. Also, using the fusion transcript plot function, it is possible to generate a collapsed representation of all exons that potentially are a part of a specific fusion transcript. However, I do agree that Clinker adds to the relatively small repertoire of tools that can be used for the daunting task of appreciating the complexity of fusion transcripts. Of special importance is the ability to show supporting reads of alternative fusion variants, not necessarily reported by the fusion finder. The authors use this feature to nicely show the presence of novel breakpoints within the P2RY8-CRLF2 fusion gene in B-ALL.2. The authors write that they have confirmed the presence of the P2RY8-CRLF2 fusion isoforms with PCR and Sanger sequencing. However, I do not see this supporting data. Additionally, the authors have performed functional experiments and find that a novel fusion isoform of P2RY8-CRLF2 drive CRLF3 overexpression in BaF3 cells. However, I am missing the supporting data behind these conclusions. In addition to the resulting flow cytometry data, I would have liked to see a sequence confirmation of the cloned canonical and alternative variants of P2RY8-CRLF2 and a validation of the resulting constitutive expression of these isoforms in transduced BaF3 cells.3. Some suggestions to the tool: For the plots output by Gviz, could the transcripts originating from each gene be colored in separate colors to distinguish the different parts of the fusion transcript? I also miss some information of the original genomic location of the partner genes, for instance the chromosome and start/stop coordinates.4. I cannot see a reference or accession number to where (or if) the RNA-seq data from the six B-cell ALL patient samples used in the manuscript have been deposited. According to GigaScience's standards for data sharing, this should be included and/or excerpts of the data could be used as example data for the tool.Minor comments:- The quality of the figures in the manuscript is somewhat poor and should be improved. Especially for figure 2, the resolution and the size of the IGV screenshots should be increased.- Nomenclature: When referring to the P2RY8-CRLF2 fusion, there is a mix of using italics and regular throughout the manuscript. Italics should be used when referring to the fusion on a gene level and regular font when referring to proteins (e.g. line 15: page 9) - A reference to the original discovery of the BCR-ABL1 oncogene should be added (line 31,32: page 1). - I might be missing this, but from the manuscript and the

Github wiki, I can't find an option to run Clinker to visualize all nominated fusion transcripts in an input file. Only the option to input each fusion name in the "fusions" parameter. In the manuscript, under software requirements, a benchmarking of running 2007 fusion genes is mentioned. I think this is of importance, as this is a nice feature of Clinker; to be able to visualize many fusion genes rapidly, for exploration of fusion gene predictions that are often hampered by false positives.- In the Github wiki, the parameter to assign which columns contains chromosome and coordinate of breakpoints is sometime referred to as "pos" and sometimes as "col". It seems to me that only "col" works as I get an error when running with "pos".

Level of Interest

Please indicate how interesting you found the manuscript: An article of importance in its field

Quality of Written English

Please indicate the quality of language in the manuscript: Acceptable

Declaration of Competing Interests

Please complete a declaration of competing interests, considering the following questions:

- Have you in the past five years received reimbursements, fees, funding, or salary from an organisation that may in any way gain or lose financially from the publication of this manuscript, either now or in the future?
- Do you hold any stocks or shares in an organisation that may in any way gain or lose financially from the publication of this manuscript, either now or in the future?
- Do you hold or are you currently applying for any patents relating to the content of the manuscript?
- Have you received reimbursements, fees, funding, or salary from an organization that holds or has applied for patents relating to the content of the manuscript?
- Do you have any other financial competing interests?
- Do you have any non-financial competing interests in relation to this paper?

If you can answer no to all of the above, write 'I declare that I have no competing interests' below. If your reply is yes to any, please give details below.

I am a co-author of Lågstad,S. (2017) Chimeraviz: Visualization tools for gene fusions.

I agree to the open peer review policy of the journal. I understand that my name will be included on my report to the authors and, if the manuscript is accepted for publication, my named report including any attachments I upload will be posted on the website along with the authors' responses. I agree for my report to be made available under an Open Access Creative Commons CC-BY license (http://creativecommons.org/licenses/by/4.0/). I understand that any comments which I do not wish to

be included in my named report can be included as confidential comments to the editors, which will not be published.

I agree to the open peer review policy of the journal

To further support our reviewers, we have joined with Publons, where you can gain additional credit to further highlight your hard work (see: https://publons.com/journal/530/gigascience). On publication of this paper, your review will be automatically added to Publons, you can then choose whether or not to claim your Publons credit. I understand this statement.
