# Supplementary material for: Clinker: visualizing fusion genes detected in RNA-seq data
Source: Gigascience. 2018 Jul 4;7(7):giy079. doi: 10.1093/gigascience/giy079 (PMC6065480; doi:10.1093/gigascience/giy079)
Supplement: Reviewer_1_Report_(Revision_1) -- Andreas Hoff — 5/24/2018 Reviewed [file giy079_reviewer_1_report_(revision_1).pdf]

Reviewer Report

Title: **Clinker: visualising fusion genes detected in RNA-seq data**

Version: **Revision 1** **Date:** 5/24/2018

Reviewer name: **Andreas Hoff**

Reviewer Comments to Author:

By revising the manuscript, the authors have slightly improved the overall description of the useful fusion visualization tool; Clinker. Most of the points I raised have been satisfactory answered and accounted for. The paper provides an excellent description of the Clinker software and demonstrates its application with an example of the P2RY8-CRLF2 fusion and detection of multiple fusion transcript isoforms in B-ALL. However, I am still missing some supporting data for the functional experiments and conclusions.In response to my previous comment #2, the authors added Sanger sequencing data confirming the presence of three alternative breakpoints of P2RY8-CRLF2 in patient 6. However, I also asked about Sanger sequencing data confirming the cloned vectors containing canonical, alternate and frameshift PCR products before being transduced into BaF3 cells. The authors should have this data, as they in the methods write: "PCR products were cloned into P-GEM-T easy vector (Promega), Sanger sequenced and then subcloned into a retroviral pMSCV-GFP retroviral expression vector". This information would further support the flow cytometry data and provide credibility that we are actually seeing overexpression of CRLF2 as a result of the expression of the canonical and alternate fusion transcripts. Especially since the alternate and frameshift fusions were cloned using the same primers. The data can easily be added as a subpanel to supplementary figure 4.In addition, I am wondering if the authors know what the longer band (~620bp) and RT-PCR product is in supplementary figure S3 and if this is backed up by RNA-seq data and visualization by Clinker?Some minor comments:- Please make sure you refer to each supplementary item in the text. I cannot see a reference to supplementary figure 1. Also the added supplementary figure 3 should be referred to on page 9, line 10.- The reference to supplementary figure 3 on line 29/30 should be changed to supplementary figure 4.- In the figure legend for figure 3 you refer to supplementary tables 1 and 2. However, I can only see one supplementary table.- In the caption of figure S3 your write "DNA gel..". The gel is showing RT-PCR products confirming the presence of fusion transcripts at the RNA level.

Level of Interest

Please indicate how interesting you found the manuscript: Choose an item.

Quality of Written English

Please indicate the quality of language in the manuscript: Choose an item.

Declaration of Competing Interests

Please complete a declaration of competing interests, considering the following questions:

- Have you in the past five years received reimbursements, fees, funding, or salary from an organisation that may in any way gain or lose financially from the publication of this manuscript, either now or in the future?

- Do you hold any stocks or shares in an organisation that may in any way gain or lose financially from the publication of this manuscript, either now or in the future?
- Do you hold or are you currently applying for any patents relating to the content of the manuscript?
- Have you received reimbursements, fees, funding, or salary from an organization that holds or has applied for patents relating to the content of the manuscript?
- Do you have any other financial competing interests?
- Do you have any non-financial competing interests in relation to this paper?

If you can answer no to all of the above, write 'I declare that I have no competing interests' below. If your reply is yes to any, please give details below.

I declare that I have no competing interests

I agree to the open peer review policy of the journal. I understand that my name will be included on my report to the authors and, if the manuscript is accepted for publication, my named report including any attachments I upload will be posted on the website along with the authors' responses. I agree for my report to be made available under an Open Access Creative Commons CC-BY license (http://creativecommons.org/licenses/by/4.0/). I understand that any comments which I do not wish to be included in my named report can be included as confidential comments to the editors, which will not be published.

I agree to the open peer review policy of the journal

To further support our reviewers, we have joined with Publons, where you can gain additional credit to further highlight your hard work (see: https://publons.com/journal/530/gigascience). On publication of this paper, your review will be automatically added to Publons, you can then choose whether or not to claim your Publons credit. I understand this statement. Yes
